# Hydrogen peroxide mediates high-intensity blue light-induced hypocotyl phototropism of cotton seedlings

**DOI:** 10.1007/s44154-023-00111-3

**Published:** 2023-07-26

**Authors:** Qian-yi Lv, Qing-ping Zhao, Chen Zhu, Meichen Ding, Fang-yuan Chu, Xing-kun Li, Kai Cheng, Xiang Zhao

**Affiliations:** 1 National Key Laboratory of Cotton Bio-Breeding and Integrated Utilization, School of Life Sciences, Henan University, Kaifeng, 475004 China; 2 College of Life Science and Agricultural Engineering, Nanyang Normal University, 1638 Wolong Road, Nanyang, 473061 Henan China

**Keywords:** Hydrogen peroxide, High-intensity blue light, Hypocotyl phototropism, Cotton

## Abstract

Phototropism is a classic adaptive growth response that helps plants to enhance light capture for photosynthesis. It was shown that hydrogen peroxide (H_2_O_2_) participates in the regulation of blue light-induced hypocotyl phototropism; however, the underlying mechanism is unclear. In this study, we demonstrate that the unilateral high-intensity blue light (HBL) could induce asymmetric distribution of H_2_O_2_ in cotton hypocotyls. Disruption of the HBL-induced asymmetric distribution of H_2_O_2_ by applying either H_2_O_2_ itself evenly on the hypocotyls or H_2_O_2_ scavengers on the lit side of hypocotyls could efficiently inhibit hypocotyl phototropic growth. Consistently, application of H_2_O_2_ on the shaded and lit sides of the hypocotyls led to reduced and enhanced hypocotyl phototropism, respectively. Further, we show that H_2_O_2_ inhibits hypocotyl elongation of cotton seedlings, thus supporting the repressive role of H_2_O_2_ in HBL-induced hypocotyl phototropism. Moreover, our results show that H_2_O_2_ interferes with HBL-induced asymmetric distribution of auxin in the cotton hypocotyls. Taken together, our study uncovers that H_2_O_2_ changes the asymmetric accumulation of auxin and inhibits hypocotyl cell elongation, thus mediating HBL-induced hypocotyl phototropism.

**Supplementary Information:**

The online version contains supplementary material available at 10.1007/s44154-023-00111-3.

## Introduction

Phototropism is an important adaptive process induced by asymmetric light during plant growth and development. The Cholodny–Went hypothesis, which has received wide acceptance and experimental support, predicts that phototropism is caused by the asymmetric distribution of auxins (Went and Thimann 1937). Higher auxin concentrations on the shaded side promote faster elongation of epidermal cells and growth toward light (Whippo and Hangarter 2006; Holland et al. 2009). Although previous studies have focused on phototropism, there has been almost no progress in elucidating molecular mechanism until the identification of phototropins (Short and Briggs 1990; Liscum and Briggs 1995; Christie et al. 1998). As blue light (BL) receptors, two forms of phototropin (phot1 and phot2) regulate hypocotyl phototropism in a fluence-dependent manner (Liscum and Briggs 1995; Sakai et al. 2000, 2001). Phot1 regulates low (0.01–1 μmol m^−2^ s^−1^) and high (> 1 μmol m^−2^ s^−1^) BL-induced phototropism, while phot2 only works under high BL (Sakai et al. 2001; Inada et al. 2004; Zhao et al. 2013).

Transmission of the phototropism signal begins with the activation of phototropins by autophosphorylation. Phototropins are plasma membrane-related proteins, which containing two N-terminal LOV domains and a C-terminal serine/threonine kinase domain (Demarsy and Fankhauser 2009). When illuminated by low-intensity blue light (LBL), a conformational change in phot1 results from a covalent bond in the LOV domain, which activates the kinase domain at the C-terminus, resulting in autophosphorylation (Christie 2007). Activated phot1 transmits downstream signals by directly phosphorylating and dephosphorylating its substrate target such as non-phototropic hypocotyl 3 (NPH3), phytochrome kinase substrate 4 (PKS4), and ATP-binding cassette B19 (ABCB19) (Motchoulski and Liscum 1999; Christie et al. 2011; Demarsy et al. 2012). These downstream signaling molecules direct the asymmetric distribution of auxin and bend to incident light in unknown ways. NPH3, root phototropism 2 (RPT2), root curling in N-naphthylphthalamic acid 1 (RCN1), and cytosolic Ca^2+^ concentration ([Ca^2+^]_cyt_) are important elements for HBL-induced phototropism (Inada et al. 2004; Lariguet et al. 2006; Tseng and Briggs 2010; Zhao et al. 2013), but the mechanism underlying high-intensity blue light (HBL) is still elusive.

Reactive oxygen species (ROS) are reactive forms of molecular oxygen, and mainly include the superoxide anion (O^−2^), singlet oxygen, hydroxyl radical and hydrogen peroxide (H_2_O_2_) (Apel and Hirt 2004). H_2_O_2_ is the most stable ROS produced by many different enzyme-catalyzed processes, including plasma membrane NADPH oxidases, peroxisomal oxidases, type III peroxidases, and other apoplastic oxidases (Forman and Torres 2002; Smirnoff and Arnaud 2019). H_2_O_2_ is removed by enzymatic and non-enzymatic antioxidant defense systems (Gill and Tuteja 2010; Lin et al. 2022; Zhang et al. 2022). ROS-scavenging enzymes, such as peroxidase (POD), catalase (CAT), and superoxide dismutase (SOD), form an enzymatic defense system (Lin et al. 2022). Two non-enzymatic antioxidants, ascorbic acid (AsA) and glutathione (GSH), produced by the AsA-GSH cycle, constitute the non-enzymatic antioxidant defense system that maintains the balance of redox substances in plants (Lin et al. 2022; Peng et al. 2022). H_2_O_2_ is viewed as a toxic cellular metabolite that causes oxidative damage when plants respond to different biotic or abiotic stresses (Henle and Linn 1997; Mittler 2002; Veal and Day 2011). However, beyond the traditional belief that ROS are toxic by-products of metabolic processes, increasing evidence shows that H_2_O_2_ is a translocating second messenger that interacts with other plant growth regulators, such as auxins, gibberellins, cytokinins, abscisic acid, and jasmonic acid, to mediated plant growth, development, and stress responses (Mittler et al. 2011; Li et al. 2020). Recent studies have reported that exogenous H_2_O_2_ treatment inhibits primary root gravitropism and induces root bending by regulating auxin distribution during the initial developmental stages of grass peas (Jiang et al. 2013; Zhou et al. 2018). In addition, H_2_O_2_ is directly involved in BL-induced curvature in wheat and *Gossypium arboreum* (Chandrakuntal et al. 2004; Zhu et al. 2021), but the underlying mechanism requires further investigation.

To further unravel the detailed mechanism underlying the function of H_2_O_2_ in BL-induced phototropism, both etiolated Arabidopsis hypocotyl, and larger etiolated cotton hypocotyls were used. We demonstrated that unilateral HBL-induced asymmetric distribution of H_2_O_2_ regulates phototropism by adjusting the asymmetric distribution of auxin and inhibiting cell elongation of the hypocotyl.

## Results

### H_2_O_2_ regulates HBL-induced hypocotyl phototropism

Previous studies have shown that blue light (BL)-induced accumulation of H_2_O_2_, and H_2_O_2_ directly causes coleoptile curvature in wheat (Chandrakuntal et al. 2004; Rusaczonek et al. 2021). We inferred that H_2_O_2_ acts as a downstream signaling molecule of BL to induce phototropism in etiolated cotton hypocotyls. To test this hypothesis, the BL-induced accumulation of H_2_O_2_, the effect of H_2_O_2_ on hypocotyl curvature, and the effect of H_2_O_2_ on BL-induced phototropism were investigated.

**Fig. 1:**
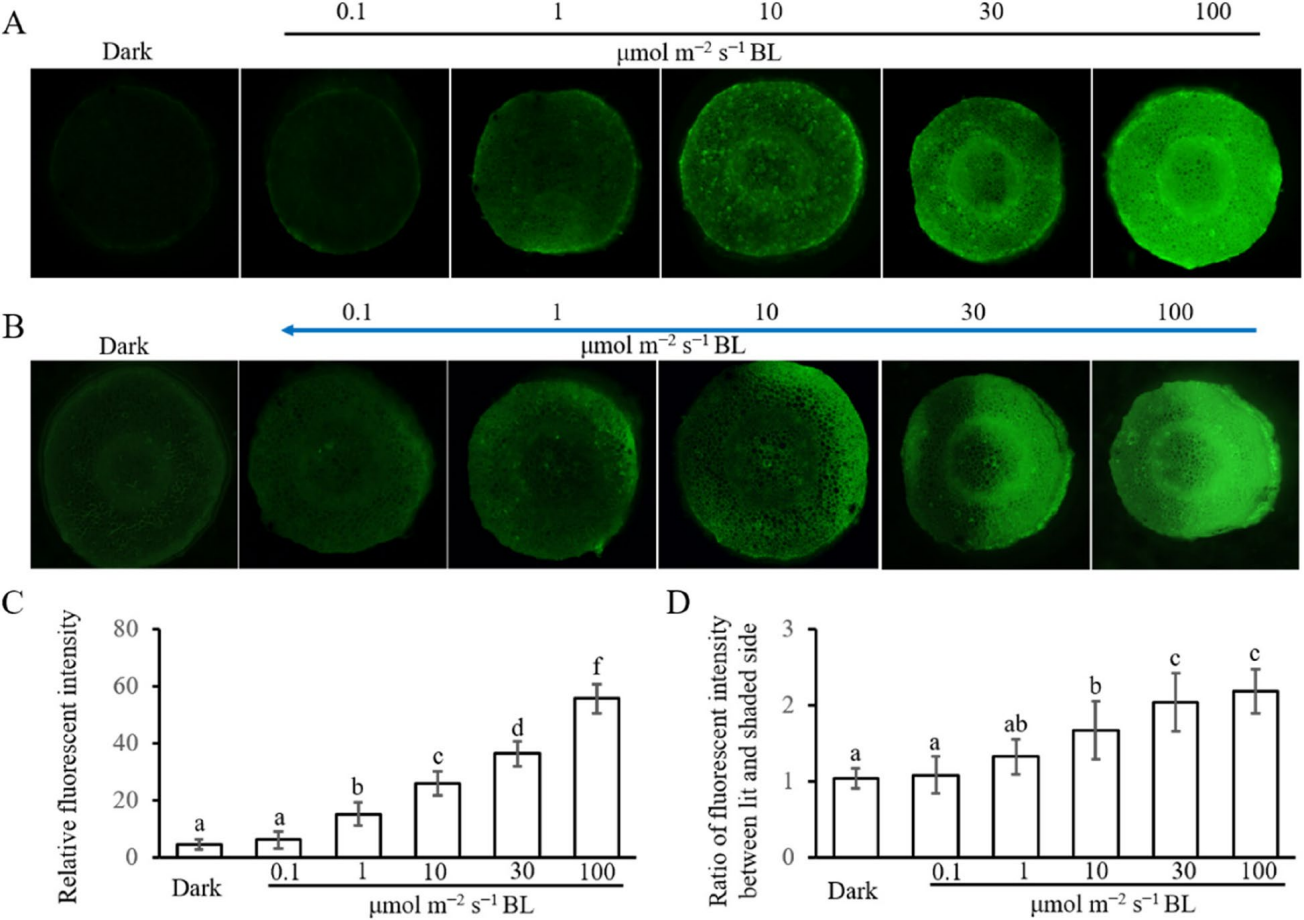
Distribution of H_2_O_2_ in response to different intensities of BL. **A** Six-day-old etiolated cotton seedlings irradiated by different intensities of BL from the top. The H_2_O_2_ was observed by using the ROS fluorescence probe DCFH-AD. **B** Six-day-old etiolated cotton seedlings irradiated by unilateral different intensities of the BL. The H_2_O_2_ was observed by using the ROS fluorescence probe DCFH-AD. The blue arrow indicates the direction of the BL. **C** Quantification of relative fluorescent intensity under condition A. Data are presented as mean ± SD (*n* ≤ 5). Statistical analysis was performed using a one-way ANOVA followed by LSD’s post-test (*P* ≤ 0.05). Small English letters indicate a difference at 0.05. **D** Quantification of relative fluorescent intensity under condition B. Data are presented as mean ± SD (*n* ≤ 5). Statistical analysis was performed using a one-way ANOVA followed by LSD’s post-test (*P* ≤ 0.05). Small English letters indicate a difference at 0.05

By using the ROS fluorescence probe DCFH-AD, we found that either irradiated from the top or side, BL of intensity 10 μmol m^−2^ s^−1^ or more can significantly induce the production of H_2_O_2_, but almost had no induction effect at an intensity less than 1 μmol m^−2^ s^−1^ (Fig. 1). According to the above experimental results, BL at an intensity of 10 μmol m^−2^ s^−1^ or more was being referred to as high-intensity blue light (HBL), and at an intensity of 1 μmol m^−2^ s^−1^ or less was referred to as low-intensity blue light (LBL).

**Fig. 2:**
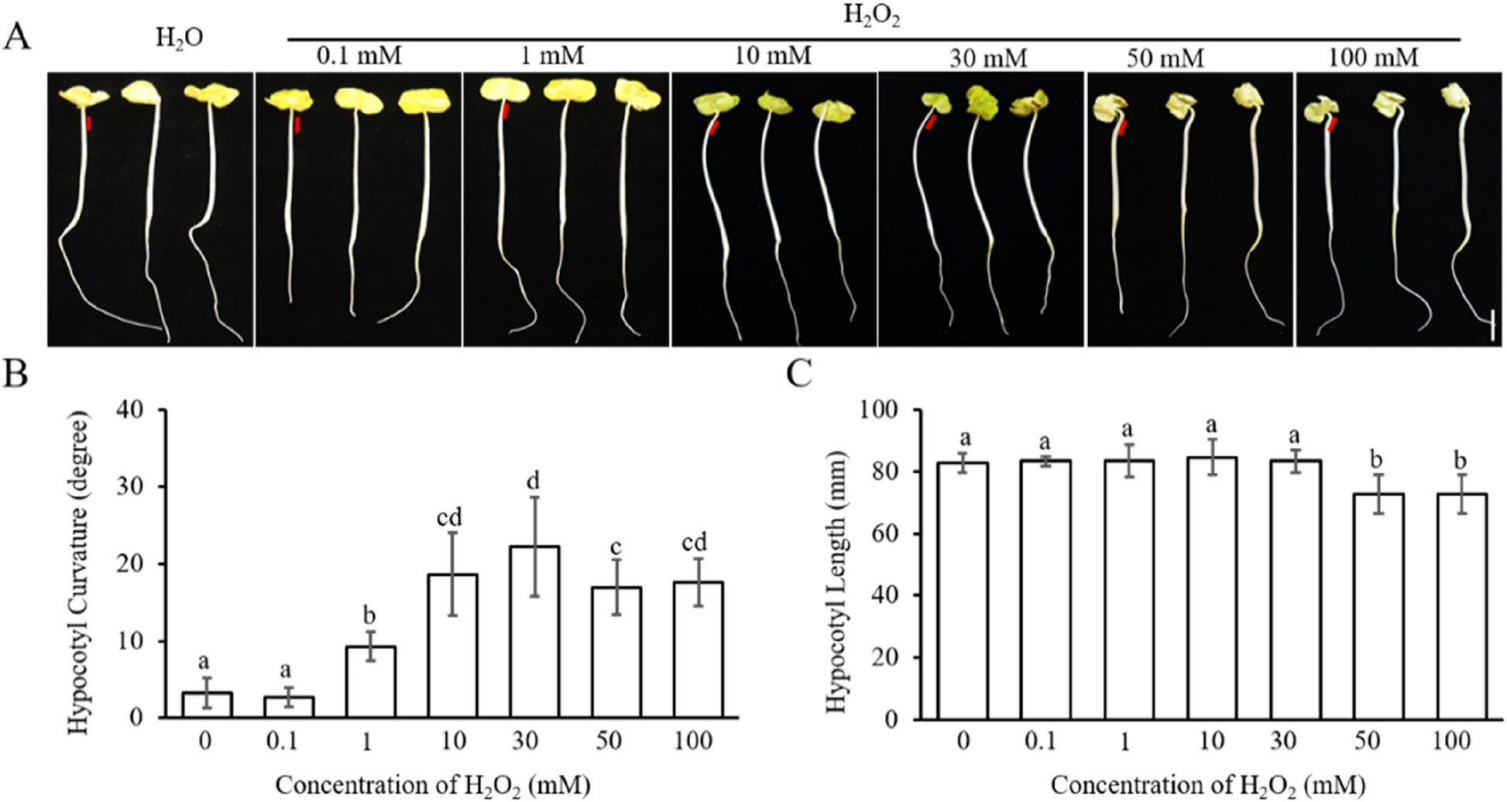
H_2_O_2_ promotes the curvature growth of the cotton hypocotyl on the smeared side in the dark. **A** Curved growth phenotype of six-day-old etiolated cotton seedlings treated with different concentrations of H_2_O_2_ in the dark. Different concentrations of H_2_O_2_ and H_2_O were applied to one side of hypocotyl. The red line represents the location where H_2_O_2_ or H_2_O be smeared. **B** Quantification of hypocotyl curvature under condition A. Data are presented as mean ± SD (*n* ≤ 20), and statistical analysis was performed using a one-way ANOVA test followed by LSD’s posttest (*P* ≤ 0.05). Small English letters indicate the difference of 0.05. **C** Quantification of hypocotyl length under condition A. Data are presented as mean ± SD (*n* ≤ 20). Statistical analysis was performed using a one-way ANOVA followed by LSD’s post-test (*P* ≤ 0.05). Small English letters indicate a difference at 0.05

When different concentrations of H_2_O_2_ were smeared onto the unilateral side of etiolated cotton, the hypocotyl bent toward the side that smeared H_2_O_2_ at concentrations greater than or equal to 1 mM. The inductive effect was extremely faint at 1 mM, and the bending curvature increased with the increase of H_2_O_2_ concentration. When the concentration was increased to 30 mM, the curvature became obvious, and the angle of the bend was approximately 22° (Fig. 2). But when the concentration was equal or greater than 50 mM, the curvature of etiolated cotton hypocotyls was greatly reduced (Fig. 2). Seedlings transferred to medium with 50 mM and 100 mM H_2_O_2_ for more than 12 h became completely soften, and the smaller curvature in these cases maybe caused by the softening of seedling. These results show that H_2_O_2_ concentration in the range of 1–30 mM was sufficient to induce tropical growth of etiolated cotton hypocotyls.

**Fig. 3:**
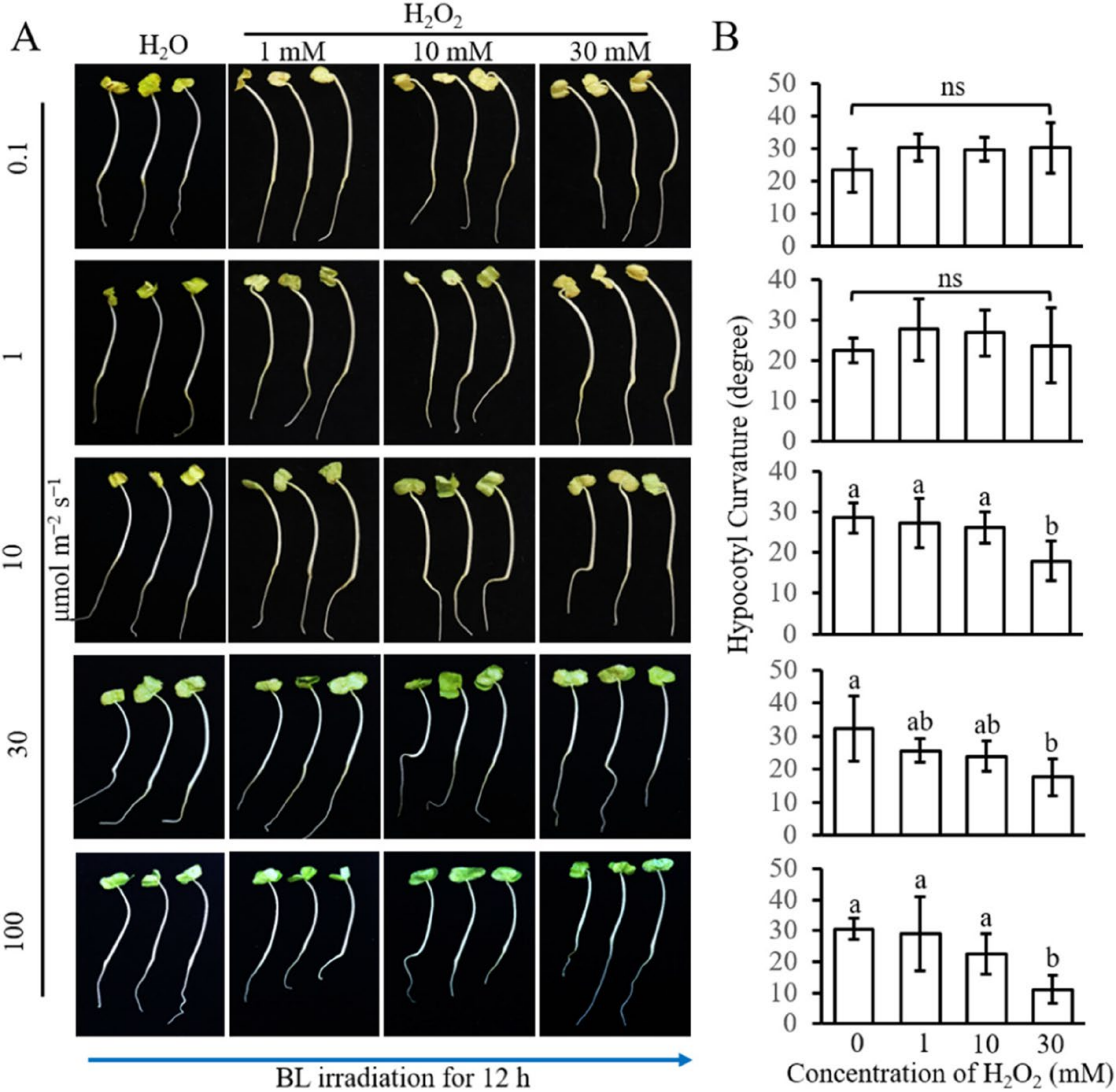
H_2_O_2_ blocks phototropic curvature of cotton hypocotyl induced by BL. **A** Phototropic phenotype of six-day-old etiolated cotton seedlings treated with different concentrations of H_2_O_2_ under different BL intensities. Solutions containing the indicated concentrations of H_2_O_2_ or H_2_O were evenly applied to the hypocotyls. The blue arrow indicates the direction of the BL. **B** Quantification of hypocotyl curvature under condition A. Each thumbnail represents one light condition. Data are presented as mean ± SD (*n* ≤ 20). Statistical analysis was performed using one-way ANOVA followed by LSD’s post-test (*P* ≤ 0.05). Small English letters indicate a difference at 0.05

Different concentrations of H_2_O_2_ solution were evenly smeared onto the hypocotyl 2 h before different intensities of BL treatment to investigate the role of H_2_O_2_ in BL-induced phototropism; H_2_O was smeared and used as a control. When irradiated with LBL for 12 h, different concentrations of H_2_O_2_ have almost no effect on the curvature of hypocotyls compared with the H_2_O treatment (Fig. 3). Under HBL conditions, the hypocotyl curvature was significantly inhibited when smeared with 30 mM H_2_O_2_ (Fig. 3). These results show that H_2_O_2_ inhibits HBL-induced phototropism in cotton, but has no effect on LBL-induced phototropism.

In addition, we found that when irradiated by unilateral HBL, the lit side of the cotton hypocotyl had stronger fluorescence intensity than the shaded side. The stronger fluorescence intensity on the lit side indicated that HBL-induced higher H_2_O_2_ accumulation on this side (Fig. 1). Further investigation is required to determine whether the function of H_2_O_2_ in HBL-induced hypocotyl phototropism is related to its asymmetric distribution.

### HBL-induced asymmetric distribution of H_2_O_2_ supports hypocotyl phototropic growth

**Fig. 4:**
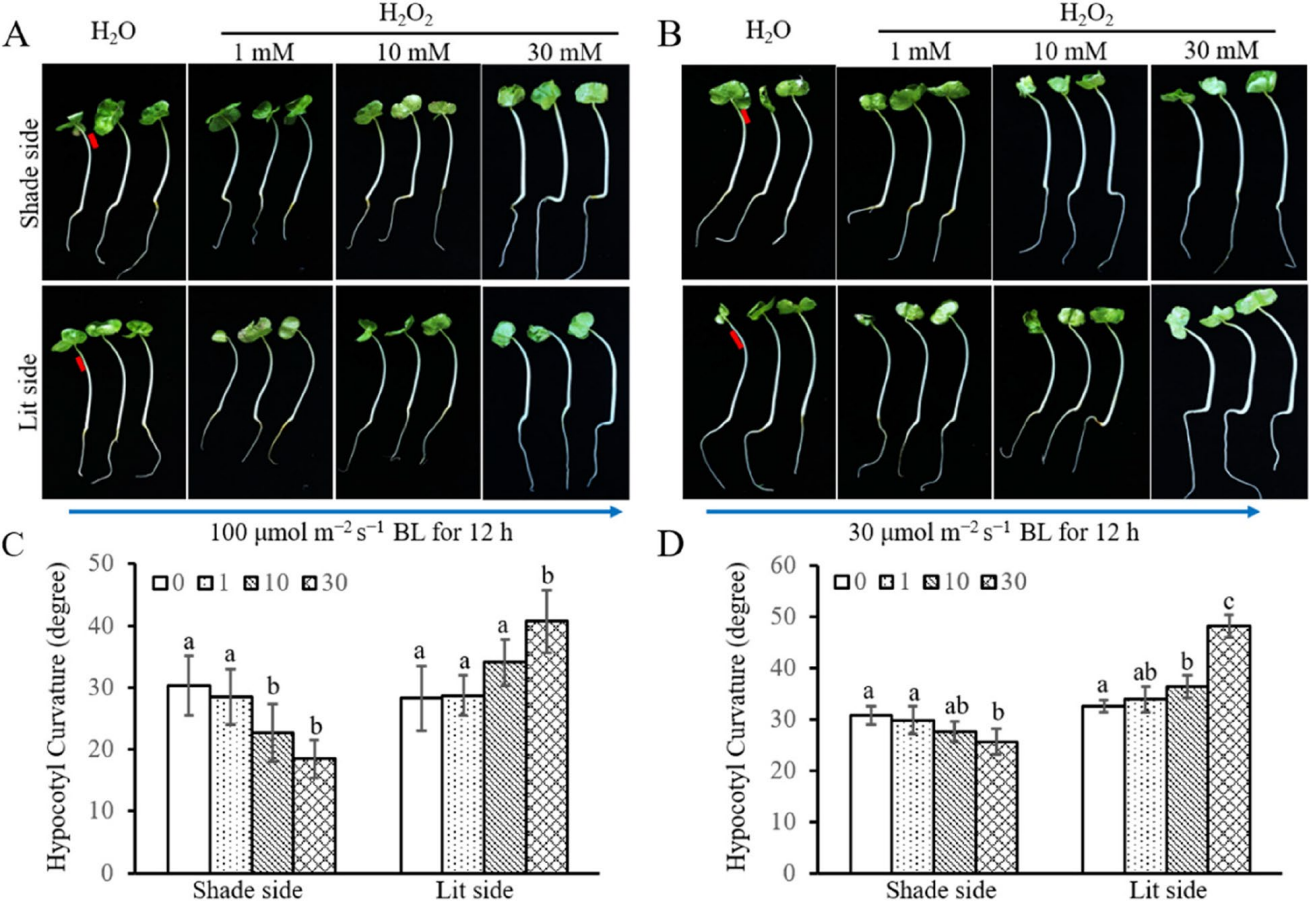
H_2_O_2_ promotes and blocks hypocotyl phototropic curvature by smearing H_2_O_2_ on the lit and shaded sides of the hypocotyl, respectively. **A** Phototropism phenotype of six-day-old etiolated cotton seedlings pretreated with different concentrations of H_2_O_2_ under 100 μmol m^−2^ s^−1^ BL. Different concentrations of H_2_O_2_ or H_2_O were smeared onto the lit or shaded side of hypocotyl. The red line represents the location where H_2_O_2_ or H_2_O be smeared. The blue arrow indicates the direction of the BL. **B** Phototropism phenotype of six-day-old etiolated cotton seedlings pretreated with different concentrations of H_2_O_2_ under 30 μmol m^−2^ s^−1^ BL. Different concentrations of H_2_O_2_ or H_2_O were smeared onto the lit and shaded sides of hypocotyls. The red line represents the location where H_2_O_2_ or H_2_O was smeared. The blue arrow indicates the direction of the BL. **C** Quantification of hypocotyl curvature under condition A. Data are presented as mean ± SD (*n* ≤ 20), and statistical analysis was performed using a one-way ANOVA test followed by LSD’s posttest (*P* ≤ 0.05). Small English letters indicate a difference at 0.05. **D** Quantification of hypocotyl curvature under condition B. Data are presented as mean ± SD (*n* ≤ 20), and statistical analysis was performed using a one-way ANOVA test followed by LSD’s posttest (P ≤ 0.05). Small English letters indicate a difference at 0.05

To verify the function of the asymmetric distribution of H_2_O_2_ in HBL-induced hypocotyl phototropism, different concentrations of H_2_O_2_ were smeared onto the lit and shaded sides of hypocotyl. Under 100 μmol m^−2^ s^−1^ unilateral HBL, 10 mM of H_2_O_2_ inhibited phototropism when applied to the shaded side, and 30 mM of H_2_O_2_ promoted phototropism when applied to the lit side (Fig. 4A and C). Under 30 μmol m^−2^ s^−1^ unilateral HBL, 30 mM of H_2_O_2_ inhibited phototropism when applied to the shaded side, and 10 mM of H_2_O_2_ promoted phototropism when applied to the lit side (Fig. 4B and D). Although H_2_O_2_ exhibited similar function under 30 and 100 μmol m^−2^ s^−1^ unilateral HBL, a higher concentration (≥ 30 mM) was needed for the inhibiting effect on the shaded side and a lower concentration (≤ 10 mM) was need for the facilitating effect on the lit side under 100 μmol m^−2^ s^−1^ unilateral HBL (Fig. 4).

**Fig. 5:**
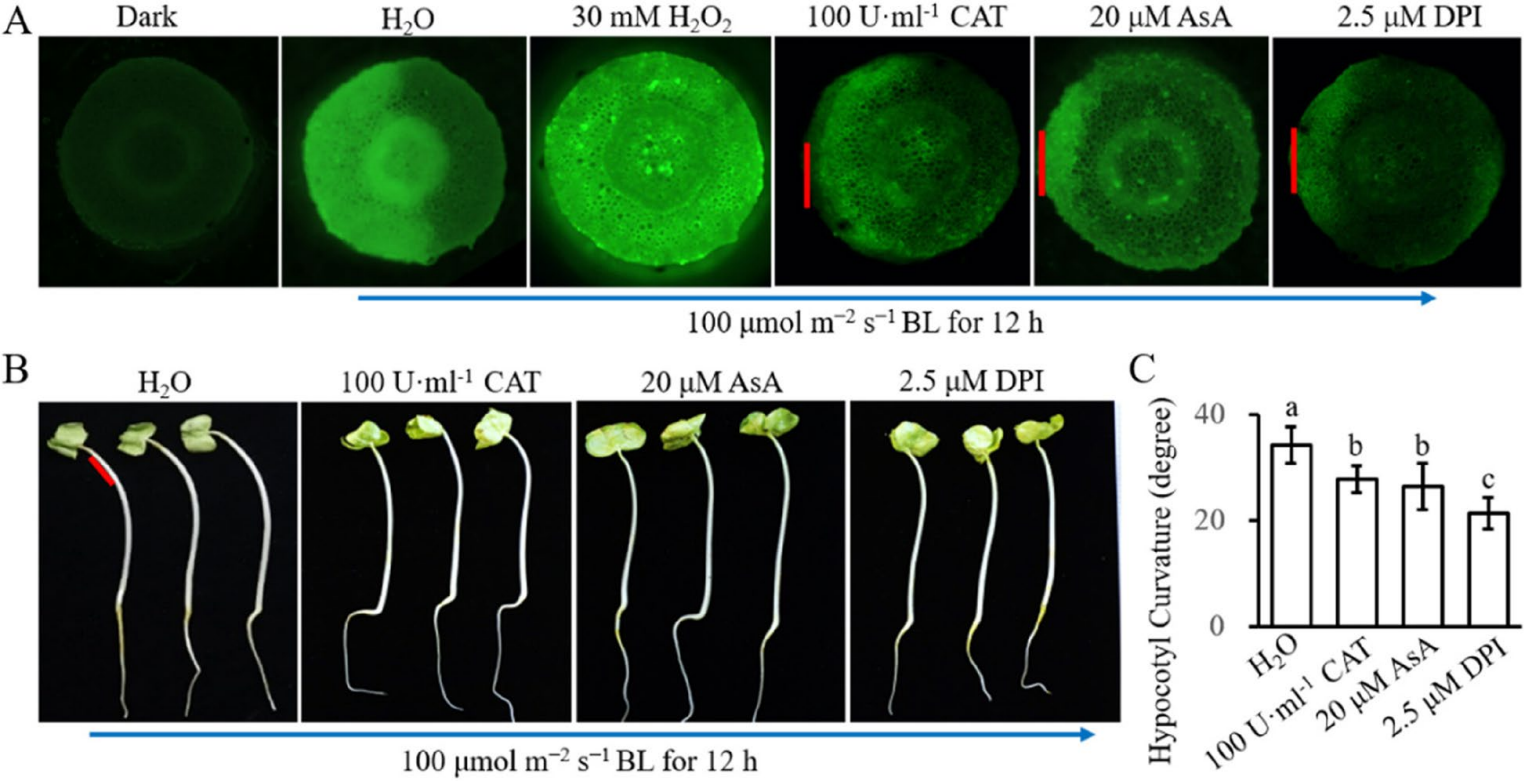
Reducing HBL-induced the asymmetrical distribution of H_2_O_2_ blocks phototropic curvature of hypocotyl **A** Distribution of H_2_O_2_ in six-day-old etiolated cotton seedlings that pretreated with 30 mM H_2_O_2_ evenly or100 U·ml^−1^ CAT, 20 μM AsA or 2.5 μM DPI in the lit side 2 h before exposure to unilateral 100 μmol m^−2^ s^−1^ BL. The red line represents the location where CAT, AsA, and DPI be smeared. The blue arrow indicates the direction of the BL. **B** Phototropism phenotype of six-day-old etiolated cotton seedlings pretreated with 100 U·ml^−1^ CAT, 20 μM AsA or 2.5 μM DPI in the lit side 2 h before exposure to unilateral 100 μmol m^−2^ s^−1^ BL. The red line represents the location where CAT, AsA, and DPI be smeared. The blue arrow indicates the direction of the BL. **C **Quantification of hypocotyl curvature under condition A. Data are presented as mean ± SD (*n* ≤ 20), statistical analysis was performed using a one-way ANOVA test followed by LSD’s posttest (*P* ≤ 0.05). Small English letters indicate a difference at 0.05

To further prove the effect of H_2_O_2_ on HBL-induced hypocotyl phototropism, the H_2_O_2_ scavengers catalase (CAT), ascorbic acid (AsA) and diphenyleneiodonium chloride (DPI) were used. CAT is ROS-scavenging enzyme that participates in removing H_2_O_2_ (Gill and Tuteja 2010; Lin et al. 2022). AsA is an important antioxidant compound used for H_2_O_2_ scavenging (Lin et al. 2022; Peng et al. 2022). DPI is a specific inhibitor of NADPH oxidase, which promotes an increase in H_2_O_2_ (Smirnoff and Arnaud 2019). When smeared to the lit side of the hypocotyl, CAT, AsA and DPI reduce the HBL-induced phototropic curvature (Fig. 5A and B).

Using the ROS fluorescence probe DCFH-AD, we found that the HBL-induced asymmetric distribution of H_2_O_2_ was disturbed by exogenous 30 mM H_2_O_2_ evenly smeared to the hypocotyl or H_2_O_2_ scavengers CAT, AsA, and DPI smeared to the lit side, although the application of H_2_O_2_ increased the H_2_O_2_ content, the application of H_2_O_2_ scavengers CAT, AsA and DPI decreased the H_2_O_2_ content (Fig. 5C). These results indicate that the asymmetric distribution of endogenous H_2_O_2_ is necessary for unilateral HBL-induced phototropism of the hypocotyl, and interference of the asymmetric distribution of H_2_O_2_ impairs HBL-induced phototropism.

### H_2_O_2_ regulates HBL-induced phototropism by inhibiting cell elongation

**Fig. 6:**
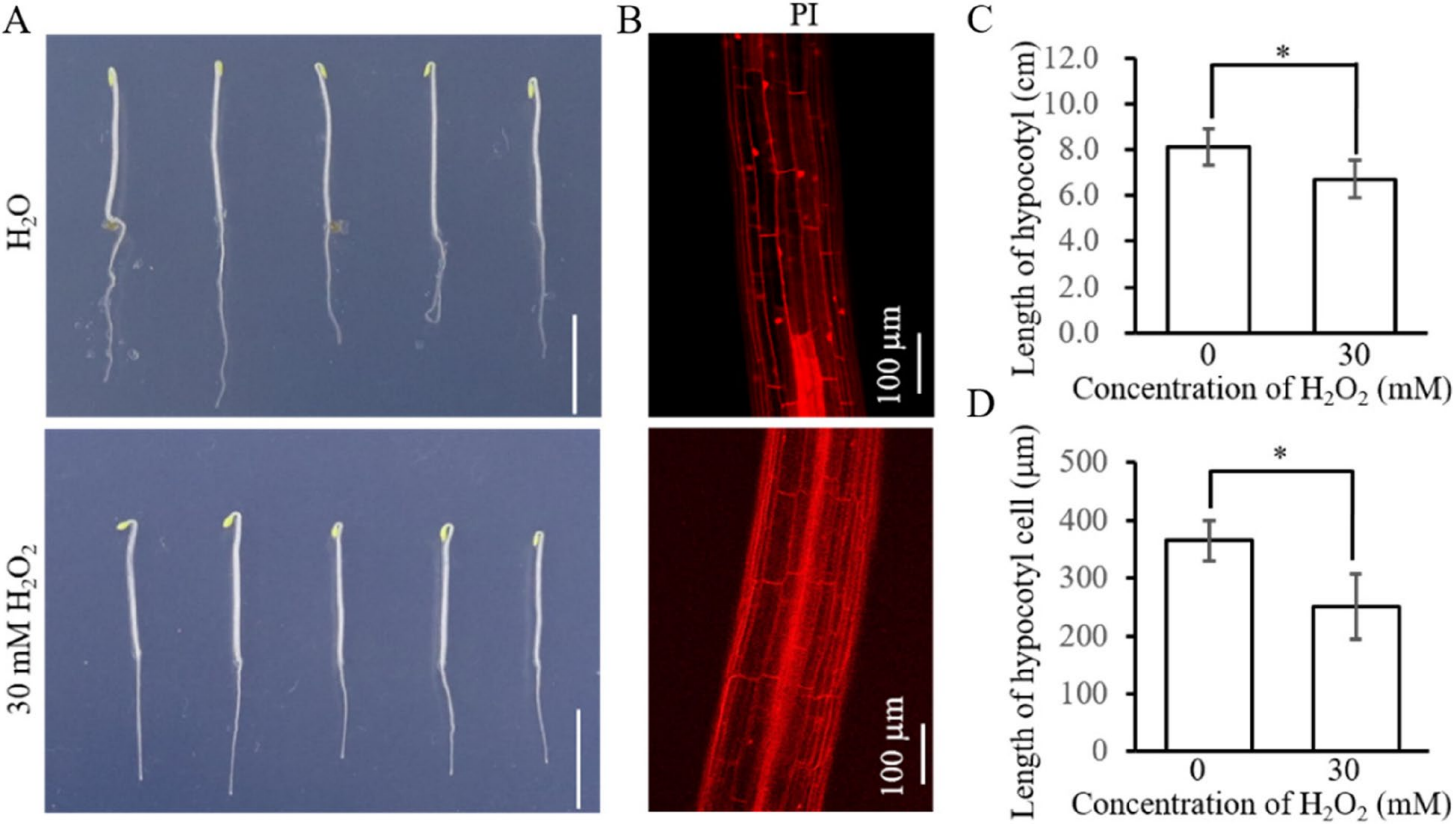
H_2_O_2_ inhibits elongation of hypocotyl and hypocotyl cell of etiolated Arabidopsis. Three-day-old etiolated Arabidopsis seedlings were transferred to a fresh medium with or without 30 mM H_2_O_2_ for 12 h. **A** The elongation phenotype of hypocotyl with or without 30 mM H_2_O_2_ treatment for 12 h under darkness. **B **The length of hypocotyl cells was observed under a microscope following PI staining in color mode. Bar = 100 µm. **C** Quantification of hypocotyl length under condition A. Data are presented as mean ± SD (*n* ≤ 15). Statistical analysis was performed using Student’s *t*-test, and significant differences are indicated by asterisks (*, *P* ≤ 0.05). **D** Quantification of hypocotyl cell length under condition B. Data are presented as mean ± SD (*n* ≤ 15), and statistical analysis was performed by using Student’s *t*-test, and significant differences are indicated by asterisks (*, *P* ≤ 0.05)

Phototropism is caused by the faster elongation of cells in the shaded side compared to the lit side (Whippo and Hangarter 2006; Christie et al. 2011). Our results showed that H_2_O_2_ inhibited phototropism when applied to the shaded side, but promoted phototropism when applied to the lit side under HBL (Fig. 4), indicating that the effect of H_2_O_2_ exerts its function on HBL-induced phototropism is dependent on its negative effect on growth. We detected the effect of H_2_O_2_ on hypocotyl elongation and found that evenly smeared 30 mM H_2_O_2_ on hypocotyl of Arabidopsis reduced the length of hypocotyl as well as hypocotyl cells in darkness (Fig. 6). Consistently, 30 mM H_2_O_2_ reduced the increased length of etiolated cotton hypocotyls, and the degree of reduction became increasingly obvious with elongated treated time (Supplementary Fig. S1). These results show that H_2_O_2_ regulates HBL-induced phototropism dependent on its inhibitory effect on hypocotyl cell elongation.

### H_2_O_2_ impaired HBL-induced asymmetrical distribution of auxin

**Fig. 7:**
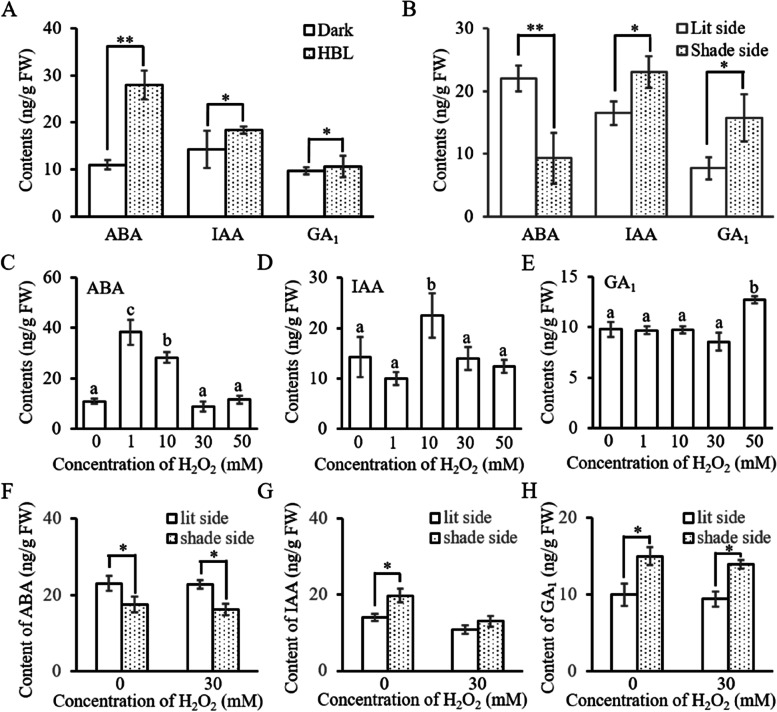
H_2_O_2_ inhibits HBL-induced asymmetrical distribution of IAA in cotton hypocotyl. HBL represents 100 μmol m^−2^ s^−1^ unilateral BL, and different concentrations of H_2_O_2_ were evenly smeared on the hypocotyl. **A** HBL increases the expression of ABA, IAA, and GA_1_. Data are presented as mean ± SD (*n* ≤ 6). Statistical analysis was performed by using Student’s *t*-test, and significant differences are indicated by asterisks (*, *P* ≤ 0.05; **, *P* ≤ 0.01). **B** HBL induces asymmetrical distribution of ABA, IAA, and GA_1_. Data are presented as mean ± SD (*n* ≤ 6). Statistical analysis was performed using Student’s *t*-test, and significant differences are indicated by asterisks (*, *P* ≤ 0.05; **, *P* ≤ 0.01). Effects of different concentrations of H_2_O_2_ on the level of ABA (**C)** IAA (**D**) and GA_1_ (**E)** Data are presented as mean ± SD (*n* ≤ 6). Statistical analysis was performed using a one-way ANOVA test followed by LSD’s posttest (*P* ≤ 0.05). Effects of different concentrations of H_2_O_2_ on HBL-induced asymmetrical distribution of ABA (**F)** IAA (**G)** and GA_1 _(**H**) Data are presented as mean ± SD (*n* ≤ 6), statistical analysis was performed using Student’s *t*-test, and significant differences are indicated by asterisks (*, *P* ≤ 0.05)

H_2_O_2_ is a significant regulatory component that interacts with other plant growth regulators, such as auxins, gibberellins (GA), cytokinins, abscisic acid (ABA), jasmonic acid (JA), ethylene, salicylic acid (SA), and nitric oxide (NO) (Neill et al. 2002; Nazir et al. 2020). Previous studies showed that auxin, ABA and GA participated in the regulation of phototropism (Ding et al. 2011; Liscum et al. 2014; Zhao et al. 2020; Zhu et al. 2021). To investigate whether H_2_O_2_ exerted its function in HBL-induced hypocotyl phototropism by interacting with auxin, ABA, or GA, the levels and distribution of IAA, ABA, and GA_1_ were determined. The results showed that HBL-induced an increasing in and an asymmetric distribution of ABA, IAA, and GA_1_ (Fig. 7A and B). Exogenous H_2_O_2_ have changed the contents of ABA, IAA, and GA_1_, but there were no specific rules to follow (Fig. 7C to E). These results indicate that H_2_O_2_-induced changes of auxin, ABA, and GA content were not the reason for its function in HBL-mediated phototropism.

When evenly smeared onto the hypocotyl irradiated by HBL, H_2_O_2_ repressed the HBL-induced asymmetric distribution of IAA, but did not affect the asymmetric distribution of ABA and GA_1_ (Fig. 7F to H). In addition, evenly smeared H_2_O_2_ on the hypocotyl impaired the asymmetric distribution of H_2_O_2_ (Fig. 5). These results indicate that the asymmetric distribution of H_2_O_2_ guaranteed the HBL-induced asymmetric distribution of auxin, and the interference of the asymmetric distribution of H_2_O_2_ impaired auxin accumulation on shaded side.

**Fig. 8:**
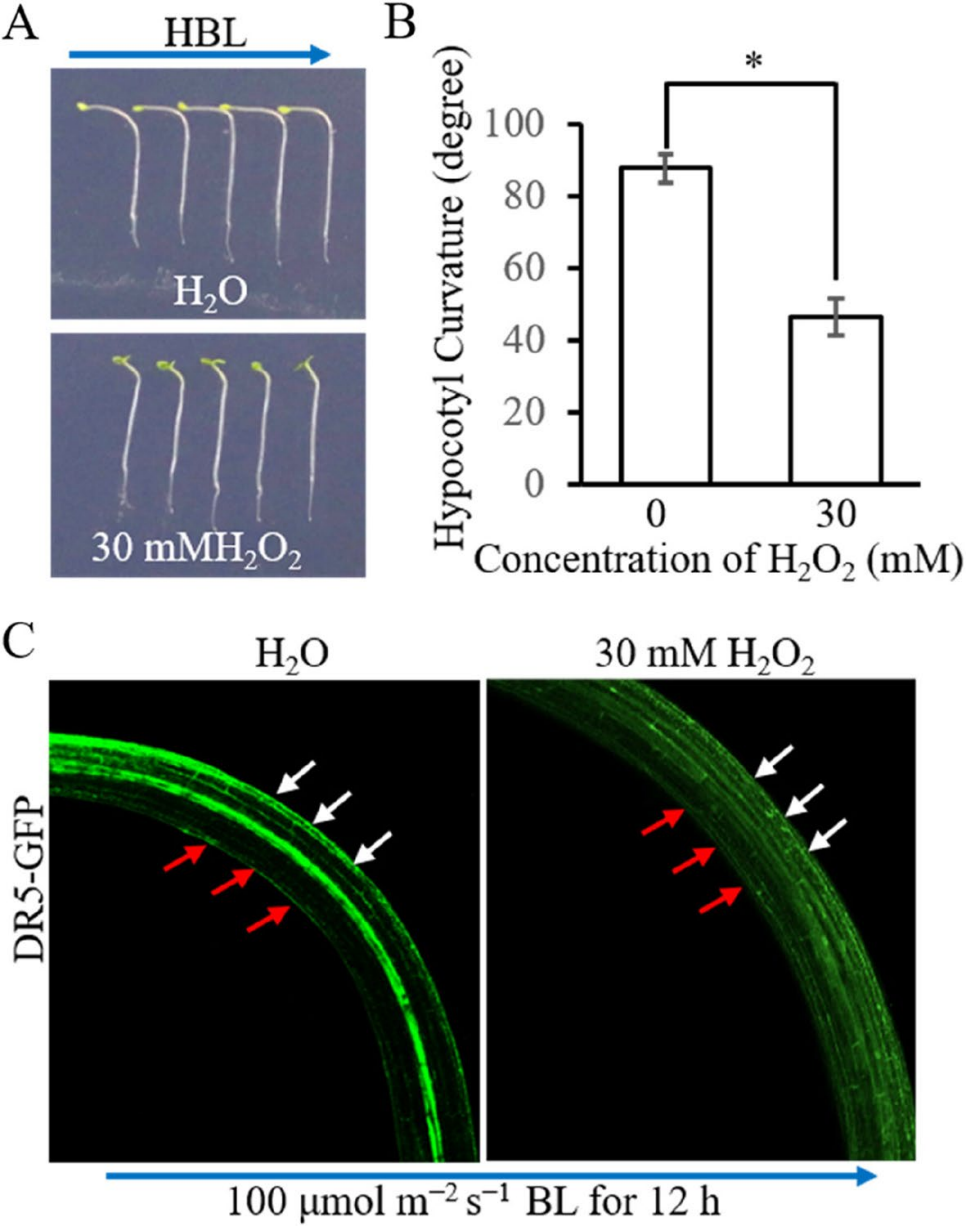
H_2_O_2_ disrupts HBL-induced asymmetrical distribution of auxin in Arabidopsis hypocotyl. Three-day-old etiolated Arabidopsis seedlings were transferred to fresh medium with or without 30 mM H_2_O_2_ for 2 h before exposure to unilateral 100 μmol m^−2^ s^−1^ BL. **A** Phototropism phenotype of Arabidopsis seedlings after irradiated by unilateral 100 μmol m^−2^ s^−1^ BL for 12 h. The blue arrow indicates the direction of the HBL. **B** Quantification of hypocotyl curvature under condition A. Data are presented as mean ± SD (*n* ≤ 20), statistical analysis was performed by using Student’s* t*-test, and significant differences are indicated by asterisks (*, *P* ≤ 0.05). **C** Effect of exogenous H_2_O_2_ on the auxin distribution monitored by DR5-GFP reporter under 100 μmol m^−2^ s^−1^ BL. Three-day-old etiolated Arabidopsis seedlings expressing DR5-GFP were used in this study. The red and white arrows indicate the lit and shaded side of the hypocotyl. The blue arrow indicates the direction of 100 μmol m^−2^ s^−1^ BL

Wild-type Arabidopsis seedlings expressing the auxin reporter gene DR5-GFP were used to directly observe the effect of H_2_O_2_ on the phototropism and auxin distribution. The results showed that exogenous H_2_O_2_ lowered the curvature of HBL-induced phototropism and the asymmetrical distribution of auxin (Fig. 8). These results indicate that H_2_O_2_ regulated HBL-induced phototropism by disturbing the asymmetrical distribution of auxin.

**Fig. 9:**
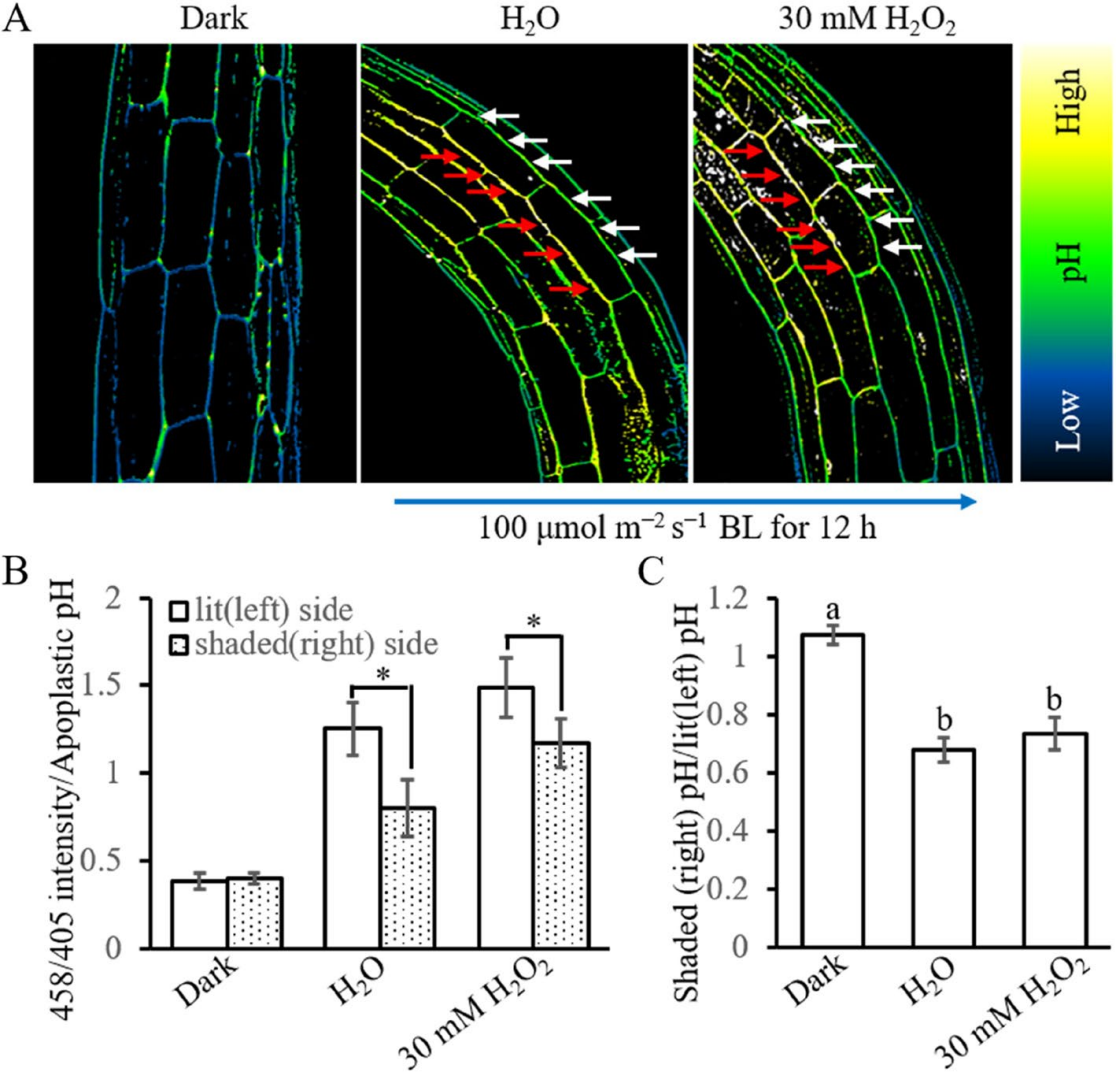
Effect of H_2_O_2_ on HBL-induced apoplastic pH difference between lit and shade side. Three-day-old etiolated Arabidopsis seedlings were transferred to fresh medium with or without 30 mM H_2_O_2_ 2 h before exposure to unilateral 100 μmol m^−2^ s^−1^ BL. **A **The apoplastic pH which indicated by the relative ratio 458/405 was visualized by HPTS staining under dark or irradiated by 100 μmol m^−2^ s^−1^ unilateral BL for 12 h. The red and white arrows indicate lit and shaded sides of the hypocotyls. The blue arrow indicates the direction of 100 μmol m^−2^ s^−1^ BL. **B** Quantification of apoplastic pH (or 458/405 intensity) under condition A. Data are presented as mean ± SD (*n* ≤ 5), statistical analysis was performed by using Student’s *t*-test, and significant differences are indicated by asterisks (*, *P* ≤ 0.05). **C** Quantification of pH ratio between shaded (right) and lit (left) side under condition A. Data are presented as mean ± SD (*n* ≤ 5), statistical analysis was performed by using Student’s *t*-test, and significant differences are indicated by asterisks (*, *P* ≤ 0.05)

Previous studies have shown that apoplastic pH is important for establishing a lateral auxin gradient and phototropism (Hohm et al. 2014; Li et al. 2022). H_2_O_2_ crosstalks with apoplastic pH in many cases including under BL irradiation (Zhang et al. 2004; Janicka-Russak and Kabała, 2012). To determine whether H_2_O_2_ exerts its function in HBL-induced phototropism by altering apoplastic pH, eight-hydroxypyrene-1,3,6-trisulfonic acid trisodium salt (HPTS) was used as a fluorescent pH indicator to analyze the apoplastic pH of hypocotyl cells. Consistent with previous results, the apoplastic pH was lower on the shaded side than on the illuminated side under HBL (Fig. 9). Exogenous 30 mM H_2_O_2_ increased apoplastic pH in both lit and shade side the reduced the pH difference between these two sides (Fig. 9). Although the difference of pH ratio (lit pH /shaded pH) with or without H_2_O_2_ treatment was insignificant, this difference may also have helped to reduce HBL-induced curvature. These results showed that the pH changes caused by H_2_O_2_ treatment also be a reason for regulation the HBL-induced phototropism.

## Discussion

Phototropism is mainly regulated by the BL receptors phototropins (phot) 1 and 2 (Liscum and Briggs 1995; Sakai et al. 2000, 2001). Phot1 mediates a wide range of BL-induced phototropism, while phot2 mediated high-intensity blue light (HBL)-induced phototropism (Sakai et al. 2001; Inada et al. 2004; Zhao et al. 2013). The mechanism underlying the BL-induced phototropism was determined using LBL. Under unilateral LBL illumination, phot1 is activated by autophosphorylation, and activated PHOT1 phosphorylates the downstream signaling components, ABCB19 and PKS4 (Christie et al. 2011; Demarsy et al. 2012). PHOT1-induced phosphorylation leads to the deactivation of both ABCB19 and PKS4 (Goyal et al. 2013; Kami et al. 2014; Liscum et al. 2014; Schumacher et al. 2018). PKS4 is believed to link phot1 activation to auxin gradient formation by forming a protein complex with phot1 and NPH3 (Kami et al. 2014; Schumacher et al. 2018; Lopez Vazquez et al. 2023). ABCB19 is an auxin transporter that charges auxin efflux and is involved in auxin circulation and redistribution (Christie et al. 2011). Feronia (FER) is a versatile receptor kinase required for the formation of normal H^+^ and auxin lateral gradient under lateral BL irradiation. Activated phot1 interacts with and phosphorylates FER, and the phot1-FER pathway regulated phototropism by changing apoplastic acidification and the auxin gradient formation in hypocotyl (Haruta et al. 2014; Li et al. 2015, 2018, 2022). Activated phot1 also phosphorylates RPT2 at a conserved serine residue (S591), and the phosphorylation of S591 is required for phototropism (Waksman et al. 2023). Phosphorylation of serine at S7(orS5), S213, S223, S237, S467, S474 (or S476), and S722 (or S723) of NPH3 is necessary for the photosensory adaptation of phot1 signaling in the phototropic response. The dephosphorylation of pS213, pS223, and pS237 has been shown to occur in a phot1-activation-dependent manner, and the dephosphorylated NPH3 dissociates from the membrane to form aggregates in cytosol. The ubiquitination and internalization of phot1 mediated by the complex that formed between NPH3 and Cullin 3-RBX-E3 (CLR3) is essential for auxin polar transport (Tsuchida-Mayama et al. 2008; Sullivan et al. 2019; Kimura et al. 2021, 2022). These results suggest possible mechanisms linking activated photoreceptors to the asymmetrical distribution of auxin under LBL, but the signal transduction mechanism under HBL remains elusive.

NPH3, RPT2, RCN1 and [Ca^2+^]_cyt_ are necessary for HBL-induced phototropism (Inada et al. 2004; Lariguet et al. 2006; Tseng and Briggs 2010; Zhao et al. 2013). Phosphomimetic NPH3^SE^, but not unphosphorylatable NPH3^SA^, promotes phot2-dependent phototropism under HBL conditions (Kimura et al. 2022). Phot2 phosphorylates RPT2 at a conserved serine residue (S591) within the C-terminal region, and a mutation in S591 reduces the phototropic functionality of RPT2 (Waksman et al. 2023). Phot2-mediated HBL-induced increase of [Ca^2+^]_cyt_ is indispensable for HBL-induced phototropism, and increased [Ca^2+^]_cyt_ is derived from the inner store-dependent Ca^2+^ release pathway (Zhao et al. 2013). Although the factors mentioned above are involved in the regulation of HBL-induced phototropism, the redundant function between phot1 and phot2 drastically restricts the understanding of the mechanism for HBL-induced phototropism. Using heliophilic plant *Gossypium hirsutum*, which is the most widely cultivated allotetraploid cottons species worldwide (Zhang et al. 2008; Bao et al. 2011), we demonstrate that HBL-induced H_2_O_2_ is another important signaling molecule in HBL-induced phototropism.

Compared with etiolated hypocotyl of *Arabidopsis thaliana*, the large individual size of cotton allows for unilateral pharmacological treatment. The application of H_2_O_2_ evenly and H_2_O_2_ scavengers CAT, AsA, and DPI to the lit side disturbed the HBL-induced asymmetrical distribution of H_2_O_2_ and phototropism. Together with the results that unilateral HBL, but not LBL, can induce the asymmetrical distribution of H_2_O_2_, exogenous H_2_O_2_ can induce the hypocotyl of cotton to bend toward the side-smeared H_2_O_2_. We conclude that exogenous H_2_O_2_ can mimic function of endogenous H_2_O_2_, and unilateral HBL-induced asymmetrical distribution of H_2_O_2_ acts downstream of HBL to regulate HBL-mediated phototropism. Phototropism results from faster growth on the shaded side of the stem than on the lit side of the stem (Whippo and Hangarter 2006; Christie et al. 2011). H_2_O_2_ acts as a signal at low concentrations and inhibits growth at high concentrations (Černý et al. 2018). We inferred that the function of H_2_O_2_ in HBL-induced phototropism is dependent on its negative effects on hypocotyl growth. When evenly smeared on the hypocotyl, H_2_O_2_ reduces the hypocotyl lengths in cotton and Arabidopsis. PI staining showed that the shorter length of Arabidopsis hypocotyls was caused by the reduced hypocotyl cell length. These results indicate that H_2_O_2_ regulated HBL-induced phototropism is due to its inhibitory effect in cell elongation. Further investigation shows that the interference of H_2_O_2_ perturb the HBL-induced asymmetrical distribution of auxin and the difference of apoplastic pH between the shaded and lit sides. In addition, we found that the pH of medium with 30 mM H_2_O_2_ (the pH was close to 5) was lower than medium without H_2_O_2 _(the pH was close to 6). These results showed that effect of H_2_O_2_ on apoplastic pH dependent on its oxidation, but the mechanism remains to be fully elucidated. Previous studies have shown that increased ROS levels can regulate auxin transport by altering the expression of PINs genes, relocating auxin exporters, and auxin conjugation (Grunewald and Friml 2010; Tognetti et al. 2010; Yuan et al. 2013). Which auxin transporters participate in the regulation of HBL-induced phototropism needs further investigation.

## Materials and methods

### Plant material growth conditions

Cotton (*Gossypium hirsutum*) seeds germinated under humidified and dark conditions at 26–28 °C for 24 h were covered in wet filter paper and grown in a lightless growth chamber at 60% humidity and at 26–28 °C for 5 d to obtain etiolated cotton hypocotyls.

Wild-type Arabidopsis seedlings expressing DR5-GFP in a Col-0 ecotype background were used to generate etiolated seedlings. Seeds were sterilized and sown on Murashige and Skoog (MS) medium containing 1% agar, then incubated at 4 °C for 3 d, followed by irradiating with white light for 12 h to induce germination. Finally, seedlings were incubated vertically for 3 d under dark condition at 22 °C to obtain etiolated hypocotyls.

### Pharmacological treatment

To examine the role of H_2_O_2_, etiolated cotton hypocotyls were pretreated with different H_2_O_2_ concentrations (0.1, 1, 10, 30, 50, and 100 mM) for 2 h either unilaterally or via submersion. Seedlings pretreated with H_2_O were used as a control. Groups of hypocotyls were then unilaterally irradiated with BL of each indicated intensities. To examine the roles of catalase (CAT), ascorbic acid (AsA) or diphenyleneiodonium chloride (DPI), groups of cotton hypocotyls were smeared on one side with CAT (100 U·mL^−1^), AsA (20 μM), or DPI (2.5 μM) 2 h before unilateral irradiation with BL of indicated intensities.

To examine the effect of H_2_O_2_ on hypocotyl phototropism in Arabidopsis, three-day-old Col-0 containing a DR5-GFP reporter was planted on MS medium with or without H_2_O_2_ (30 mM) exposure and irradiated with HBL for 12 h.

### Measurement of phototropic curvature

The phototropic curvature of etiolated cotton and Arabidopsis hypocotyls was investigated as previously described (Zhu et al. 2021). Briefly, etiolated cotton or Arabidopsis hypocotyls treated with a pharmacological reagent or H_2_O for 2 h were unilaterally irradiated with BL of indicated intensities. After irradiation for 12 h, photographs were acquired, and hypocotyl curvature was measured using ImageJ software.

### Measurement of hypocotyl cell lengths

Three-day-old Arabidopsis seedlings were incubated in liquid growth medium supplemented with 2 μM propidium iodide (PI) for approximately 10 min. Seedlings were then mounted on microscope slides and covered with coverslips. PI fluorescence (excitation, 536 nm; emission peak, 617 nm) was detected using a Carl Zeiss LSM 710 laser scanning confocal microscope.

### DR5-GFP fluorescence observation

Three-day-old Arabidopsis seedlings were mounted on microscope slides in liquid growth medium and covered with coverslips. DR5-GFP fluorescence was observed using a Carl Zeiss LSM 710 laser scanning confocal microscope with an excitation and emission wavelengths of 488 and 525 nm, respectively.

### Detection of H_2_O_2_ using fluorescence detection probe DCFH-AD

To detect the level and localization of H_2_O_2_ in etiolated cotton hypocotyls, the bent parts of the hypocotyls were transversely cut into pieces (approximately 0.5 cm), and these pieces were incubated in a solution supplemented with active oxygen fluorescence detection probe DCFH-AD for 5–6 min. These pieces were then rinsed, mounted on microscope slides in liquid growth medium, and covered with coverslips. Fluorescence signals were observed and photographs were obtained under a stereomicroscope using excitation and emission wavelengths of 488 and 525 nm, respectively.

### Eight-hydroxypyrene-1,3,6-trisulfonic acid trisodium salt (HPTS) staining assay

 was performed as previously described (Barbez et al. 2017) with some modifications. Three-day-old Arabidopsis seedlings were incubated in liquid growth medium supplemented with 5 mM HPTS for approximately 30 min. The seedlings were subsequently mounted on a microscope slide in growth medium and covered with a coverslip. Seedling imaging was performed using a Carl Zeiss LSM 710 laser scanning confocal microscope with excitation and emission wavelengths of 405 and 514 nm, respectively, for protonated HPTS, and excitation and emission wavelengths of 458 and 514 nm, respectively, for deprotonated HPTS. Image analysis was performed using Fiji software as previously described (Barbez et al. 2017). The ratio of the 458/405 emission signal intensity was used to indicate pH changes as previously described (Xie et al. 2020). For lower pH leads to higher fluorescence emission following excitation with a 408 nm wavelength, and higher pH causes higher fluorescence after excitation at 458 nm, the 458/405 emission signal intensity increased with the increasing of apoplastic pH.

### Phytohormone measurements

To extract IAA, ABA, and GA_1_ from cotton seedlings, 0.2 g samples were ground into powder in liquid nitrogen and then incubated in 1 mL of extracting solution (Methanol: ddH_2_O: Glacial acetic acid = 80:19:1 by volume ratio) for 16 h in darkness at 4 °C。The mixed suspension was centrifuged (12,000 r/min, 4 °C) for 5 min, and 700–800 μL of each supernatant was collected into 2 mL centrifuge tubes. After drying in a Termovap sample concentrator, samples were promptly dissolved in 100 μL methanol (60%). Methanol solutions were centrifuged again (13,000 r/min, 4 °C, 1 min) to collect liquid supernatants. These supernatants were filtered at least twice through 0.22 μm membranes.

ABA, IAA, and GA_1_ were quantified using ultra-high-performance liquid chromatography-tandem mass spectrometry (UPLC-MS/MS). The samples were analyzed using a Xevo TQ-XS system (Waters, USA) equipped with an ESI ion source. Chromatographic separation was conducted using an ACQUITY UPLC HSS T3 column (2.1 × 100 mm, 1.8 µm) maintained at 40 °C. The mobile phase comprised solvent A (0.1% formic acid and water) and solvent B (methanol), with a flow rate of 0.3 mL min^−1^. The linear gradient was set as follows: 0–2 min, 2% B; 2–10 min, 80% B; 10–12 min, 80% B; 12–13 min, 2% B; 13–15 min, 2% B. The autosampler temperature was set to 4 °C and sample injection volume was 10 μL. MS data were collected in negative ion mode using multiple reaction monitoring mode. Precursor and fragment ions were ABA (m/z 263.16 -153.01), IAA (m/z 176.05 -130.10), and GA_1_ (m/z 374.1 -259.12). Data analysis was performed using the supplied spectrometer software (Masslynx v.4.2).

### Supplementary Information

**Additional file 1:** **SupplementaryFigure S1.** H_2_O_2_ inhibitselongation of hypocotyl of etiolated cotton Quantificationof increased hypocotyl length of six-day-old etiolated cotton seedlings evenlysmeared with or without 30 mM H_2_O_2_ for 12 h, 24 h or 36 h, respectively. The experiment was conducted in the dark. Data are presentedas mean ± SD (*n*≤ 20). Statistical analysis was performed by using Student’s *t*-test, and significant differences are indicated by asterisks (*, *P*≤0.05).

## Data Availability

Data sharing is not applicable to this article as no new data were created or analyzed in this study.

## Abbreviation

BL: Blue light
H_2_O_2_: Hydrogen peroxide
LBL: Low intensity blue light
HBL: High intensity blue light
