# Supplementary material for: Hydrogen peroxide mediates high-intensity blue light-induced hypocotyl phototropism of cotton seedlings
Source: Stress Biol. 2023 Jul 26;3(1):27. doi: 10.1007/s44154-023-00111-3 (PMC10442013; doi:10.1007/s44154-023-00111-3)
Supplement: Supplementary file 1 — Additional file 1: SupplementaryFigure S1. H2O2 inhibitselongation of hypocotyl of etiolated cotton Quantificationof increased hypocotyl length of six-day-old etiolated cotton seedlings evenlysmeared with or without 30 mM H2O2 for 12 h, 24 h or 36 h, respectively. The experiment was conducted in the dark. Data are presentedas mean ± SD (n≤ 20). Statistical analysis was performed by using Student’s t-test, and significant differences are indicated by asterisks (*, P≤0.05). [file 44154_2023_111_MOESM1_ESM.doc]

**Supplementary Figures**

**Supplementary Figure S1**

**
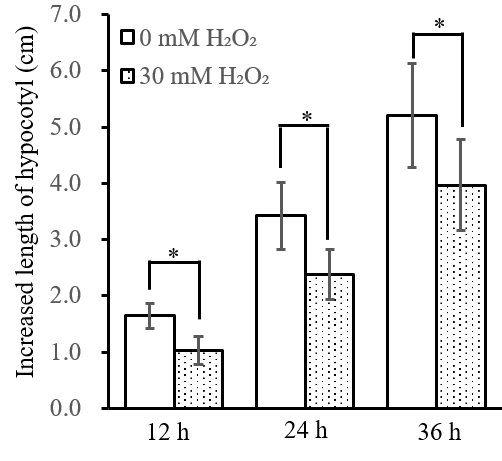
**

**Supplementary Figure S1.** H_2_O_2_ inhibits elongation of hypocotyl of etiolated cotton

Quantification of increased hypocotyl length of six-day-old etiolated cotton seedlings evenly smeared with or without 30 mM H_2_O_2_ for 12 h, 24 h or 36 h, respectively. The experiment was conducted in the dark. Data are presented as mean ± SD (n≤ 20). Statistical analysis was performed by using Student’s t-test, and significant differences are indicated by asterisks (*, P≤0.05).
